# *ADAM22* ethnic-specific variant reducing binding of membrane-associated guanylate kinases causes focal epilepsy and behavioural disorder

**DOI:** 10.1093/braincomms/fcad295

**Published:** 2023-10-27

**Authors:** Lenka Nosková, Yuko Fukata, Viktor Stránecký, Jana Šaligová, Oxana Bodnárová, Mária Giertlová, Masaki Fukata, Stanislav Kmoch

**Affiliations:** Research Unit for Rare Diseases, Department of Pediatrics and Inherited Metabolic Disorders, 1st Faculty of Medicine, Charles University in Prague, 128 08 Prague 2, Czech Republic; Division of Membrane Physiology, Department of Molecular and Cellular Physiology, National Institute for Physiological Sciences, National Institutes of Natural Sciences, Okazaki 444-8787, Japan; Department of Physiological Sciences, School of Life Science, SOKENDAI (The Graduate University for Advanced Studies), Okazaki 444-8585, Japan; Division of Molecular and Cellular Pharmacology, Nagoya University Graduate School of Medicine, Nagoya 466-8550, Japan; Research Unit for Rare Diseases, Department of Pediatrics and Inherited Metabolic Disorders, 1st Faculty of Medicine, Charles University in Prague, 128 08 Prague 2, Czech Republic; Children's Faculty Hospital, Košice 040 11, Slovakia; Children's Faculty Hospital, Košice 040 11, Slovakia; Medical Genetics Outpatient Service, Unilabs Slovakia Ltd, Košice 040 01, Slovakia; Department of Paediatric and Adolescent Medicine, Faculty of Medicine, P.J. Šafárik University,Košice 040 01, Slovak Republic; Division of Membrane Physiology, Department of Molecular and Cellular Physiology, National Institute for Physiological Sciences, National Institutes of Natural Sciences, Okazaki 444-8787, Japan; Department of Physiological Sciences, School of Life Science, SOKENDAI (The Graduate University for Advanced Studies), Okazaki 444-8585, Japan; Division of Neuropharmacology, Nagoya University Graduate School of Medicine, Nagoya 466-8550, Japan; Research Unit for Rare Diseases, Department of Pediatrics and Inherited Metabolic Disorders, 1st Faculty of Medicine, Charles University in Prague, 128 08 Prague 2, Czech Republic

**Keywords:** focal epilepsy, ethnic-specific variant, insufficient ADAM22–MAGUK interaction

## Abstract

Pathogenic variants of *ADAM22* affecting either its biosynthesis and/or its interactions with either LGI1 and/or PSD-95 have been recently identified in individuals with developmental and epileptic encephalopathy. Here, we describe a girl with seizures, delayed psychomotor development, and behavioural disorder, carrying a homozygous variant in *ADAM22* (NM_021723.5:c.2714C > T). The variant has a surprisingly high frequency in the Roma population of the Czech and Slovak Republic, with 11 of 213 (∼5.2%) healthy Roma individuals identified as heterozygous carriers. Structural *in silico* characterization revealed that the genetic variant encodes the missense variant p.S905F, which localizes to the PDZ-binding motif of ADAM22. Studies in transiently transfected mammalian cells revealed that the variant has no effect on biosynthesis and stability of ADAM22. Rather, protein–protein interaction studies showed that the p.S905F variant specifically impairs ADAM22 binding to PSD-95 and other proteins from a family of membrane-associated guanylate kinases, while it has only minor effect on ADAM22–LGI1 interaction. Our study indicates that a significant proportion of epilepsy in patients of Roma ancestry may be caused by homozygous c.2714C > T variants in *ADAM22*. The study of this *ADAM22* variant highlights a novel pathogenic mechanism of ADAM22 dysfunction and reconfirms an essential role of interaction of ADAM22 with membrane-associated guanylate kinases in seizure protection in humans.

## Teaser

Nosková *et al*. report a girl with focal epilepsy and behavioural disorder carrying homozygous variant p.S905F in ADAM22 that is frequent in Roma population. The variant causes insufficient interaction of ADAM22 to a family of membrane-associated guanylate kinases and leads to milder phenotype compared to previously reported cases.

## Graphical Abstract

**Graphical Abstract:**
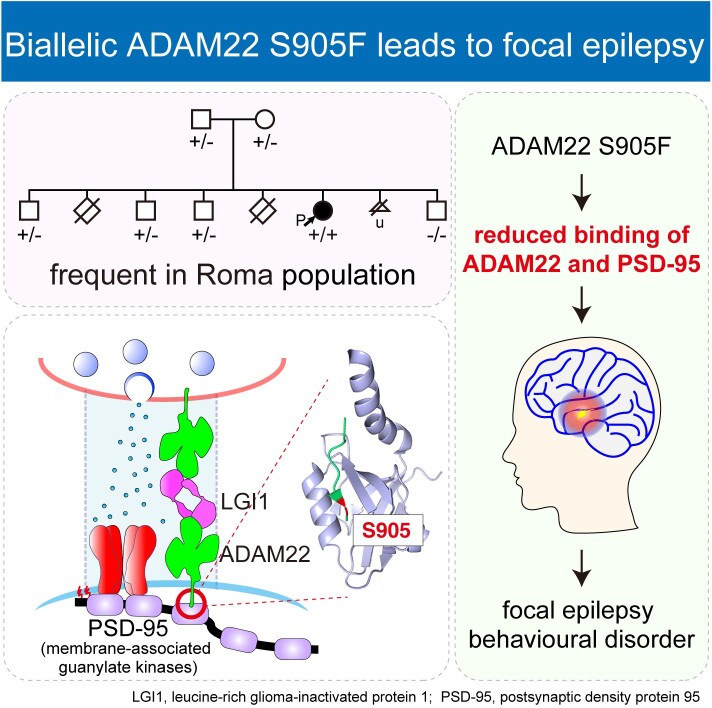

## Introduction

ADAM22 (*a d*isintegrin *a*nd *m*etalloproteinase domain-containing protein 22) is a non-catalytic metalloprotease-like protein expressed at the specialized membrane domains in neurons, such as the axon initial segment, juxtaparanodes and synapses.^1-4^ ADAM22 is a receptor for the secreted neuronal protein, LGI1 (*l*eucine-rich, *g*lioma *i*nactivated 1 protein). Together, ADAM22 and LGI1 form the hetero-tetrameric LGI1–ADAM22 complex on the neuronal cell surface, regulating synapse maturation and function in the post-natal brain.^1,5-7^ LGI1–-ADAM22 interaction is critical for AMPA-type glutamate receptor-mediated synaptic transmission and hippocampal long-term potentiation via molecular interaction with the *p*ost-synaptic *d*ensity *p*rotein PSD-95, a member of the MAGUK (*m*embrane-*a*ssociated *gu*anylate *k*inase) family.^8-10^ Variants in *LGI1* or *ADAM22* have been linked to epilepsy in humans and mouse models.^11,12^ Heterozygous pathogenic variants in *LGI1* are associated with autosomal dominant lateral temporal lobe epilepsy (OMIM # 600512).^13^ Bi-allelic pathogenic variants in *ADAM22* are associated with autosomal recessive developmental and epileptic encephalopathy (OMIM # 617933).^14-16^ All previously reported individuals with pathogenic *ADAM22* variants exhibit infantile-onset and treatment-resistant epilepsy with moderate to profound global developmental delay, hypotonia and delayed motor development. Functional studies have revealed at least three distinct pathogenic mechanisms of ADAM22 dysfunction that are dependent on the position and nature of the variant in the protein: (i) aberrant ADAM22 maturation and cellular localization; (ii) impaired ADAM22–LGI1-binding and/or (iii) disrupted interaction of the LGI1–ADAM22 complex with the PSD-95.^16^

Here, we report a girl with early-onset focal epilepsy and mild neurologic symptoms due to an ethnically-specific homozygous missense variant in the PDZ-binding domain of ADAM22 and provide functional characterization of the pathogenicity of this variant.

## Materials and methods

### Study subject and clinical examination

The proband was ascertained from a series of patients with paediatric-onset rare diseases with unknown genetic bases who underwent whole-exome sequencing (WES). Informed consent for genetic analyses was obtained for all individuals, and genetic studies were performed as approved by the Institutional Review Board of the First Faculty of Medicine of the Charles University, Prague, Czech Republic. The patient’s parents provided written informed consent for the participation in the study, clinical data and specimen collection, genetic analysis and publication of relevant findings.

### Genetic analyses

Genomic DNA extracted from leukocytes of the patient and her parents was used for WES. Exome enrichment was performed on individually barcoded samples using SeqCap EZ MedExome Probes (Roche) and sequencing was performed on Novaseq 6000 platform (Illumina) with 100 bp paired-end reads. Reads were aligned to the hg19 reference genome using Novoalign version 3.02.13 (Novocraft) with default parameters.

After genome alignment, conversion of SAM format to BAM and duplicate removal were performed using Picard Tools (2.20.8). The Genome Analysis Toolkit, GATK (3.8)^17^ was used for local realignment around indels, base recalibration, variant recalibration and variant calling. Variants were annotated using the GEMINI framework^18^ and filtered based on the population frequencies using several public databases and an in-house database of population-specific variants. Identification of candidate variants was performed for autosomal dominant (*de novo* variants) and autosomal recessive inheritance patterns. Variants were further prioritized according to the functional impact and conservation score.

Sanger sequencing was used for genotyping and segregation study of the candidate variant in family members, and functional studies in transiently transfected mammalian cells were performed. The variant in *ADAM22* was classified as pathogenic according to ACMG guidelines and submitted to ClinVar database under the accession VCV001713132.1.

### Plasmid construction

Generation of the pCAGGS: human *ADAM22*, pCAGGS: human *LGI1*–FLAG and pGW: rat *PSD-95*-FLAG were described previously.^14^  *ADAM22* S905F (NM_021723.5: c.2714C > T) was generated by the standard PCR method and the PCR product was analysed by DNA sequencing. pGW: rat *PSD-95*-GFP, pGW: rat *PSD-93*-GFP and pGW: rat *SAP102*-GFP were described previously.^10^

### Antibodies

The following antibodies were used: rabbit polyclonal antibodies to ADAM22 (aa 444-526, extracellular epitope)^14^ and GFP^10^; mouse monoclonal antibodies to ADAM22 (NeuroMab, N46/30), FLAG (Sigma-Aldrich, F3165) and PSD-95 (Thermo Fisher Scientific, MA1-046); and a guinea pig polyclonal antibody to LGI1.^19^

### Immunoprecipitation

HEK293T cells were seeded in six-well cell culture plates (5 × 10^5^ cells/well) and co-transfected for the indicated protein expression. At 24-h after transfection, the cells were washed with phosphate buffer saline (PBS) and subsequently lysed with buffer A [20 mM Tris-HCl (pH 8.0), 1 mM EDTA, 0.5% Fos-Choline-14 (Anatrace) and 50 μg/ml PMSF]. The lysates were cleared by centrifugation at 10 000 *g* for 5 min at 4°C. PSD-95–FLAG or LGI1–FLAG was immunoprecipitated with FLAG–M2 agarose (Sigma-Aldrich), washed with buffer B [20 mM Tris-HCl (pH 8.0), 1 mM EDTA, 100 mM NaCl, 1% Triton X-100 and 50 μg/ml PMSF], and eluted with buffer B containing 0.25 mg/ml FLAG peptide.^14^ For MAGUK immunoprecipitation, GFP-tagged MAGUK proteins were immunoprecipitated with anti-GFP-antibody. The immunoprecipitates were analysed by western blotting. Chemical luminescent signals were detected with a cooled CCD camera (the FUSION Solo system, Vilber-Lourmat) and the band intensities were analysed with the FUSION Capt software.

### Cell-surface binding assay

COS7 cells were seeded onto poly-*d*-lysine 12-mm cover slips in a six-well cell culture plate (2 × 10^5^ cells/well). ADAM22 was co-transfected with or without LGI1–FLAG. At 36-h after transfection, the cells were washed with DMEM and cell-surface-expressed ADAM22 or cell-surface-bound LGI1–FLAG was ‘live-labelled’ with antibodies to an extracellular epitope of ADAM22 and FLAG, respectively, by incubating COS7 cells for 30 min at 37°C. The cells were fixed with 2% paraformaldehyde/120 mM sucrose/100 mM HEPES (pH 7.4) at room temperature for 20 min and blocked with PBS containing 10 mg/ml bovine serum albumin for 10 min on ice. The fixed cells were incubated with Cy3-conjugated secondary antibody. Then, the cells were permeabilized with 0.1% Triton X-100 for 10 min, blocked with PBS containing 10 mg/ml BSA, and stained with mouse or rabbit anti-ADAM22 antibody, followed by Alexa488-conjugated secondary antibody to detect the expressed ADAM22. Total LGI1 protein expressed was also stained by FLAG antibody with Alexa 647-conjugated secondary antibody after cells were permeabilized. Then, nuclei were visualized with Hoechst dye (Thermo Fisher Scientific, 33342). Fluorescent images were taken with a confocal laser microscopy system (Carl Zeiss LSM 510).

### Statistical analysis

To perform statistical analysis, three independent experiments were included in the analyses. Results are shown as means ± SE. Statistical details of individual experiments are described in figure legends.

### Ethics declaration

Informed consent for genetic analyses was obtained for all individuals, and genetic studies were performed as approved by the Institutional Review Board of the First Faculty of Medicine of the Charles University, Prague, Czech Republic. The patient’s parents provided written informed consent for the participation in the study, clinical data and specimen collection, genetic analysis and publication of relevant findings. All data were de-identified. The study adheres to the principles set out in the Declaration of Helsinki.

## Results

### Clinical features

The female patient was born at term by spontaneous breech delivery with birth weight 3550 g and birth length 51 cm without any complications reported pre-natally and peri-natally. The developmental delay was apparent before the onset of seizure at the age of 2 years, with independent walking at 2–2.5 years, delay in speech development with first words at 2.5 years and using sentences after 3 years. She acquired diurnal toileting skills at the age of 5 years with regression after the age of six with diurnal enuresis. She had permanent nocturnal incontinence until the age 7 when the genetic examination was performed.

**Figure 1:**
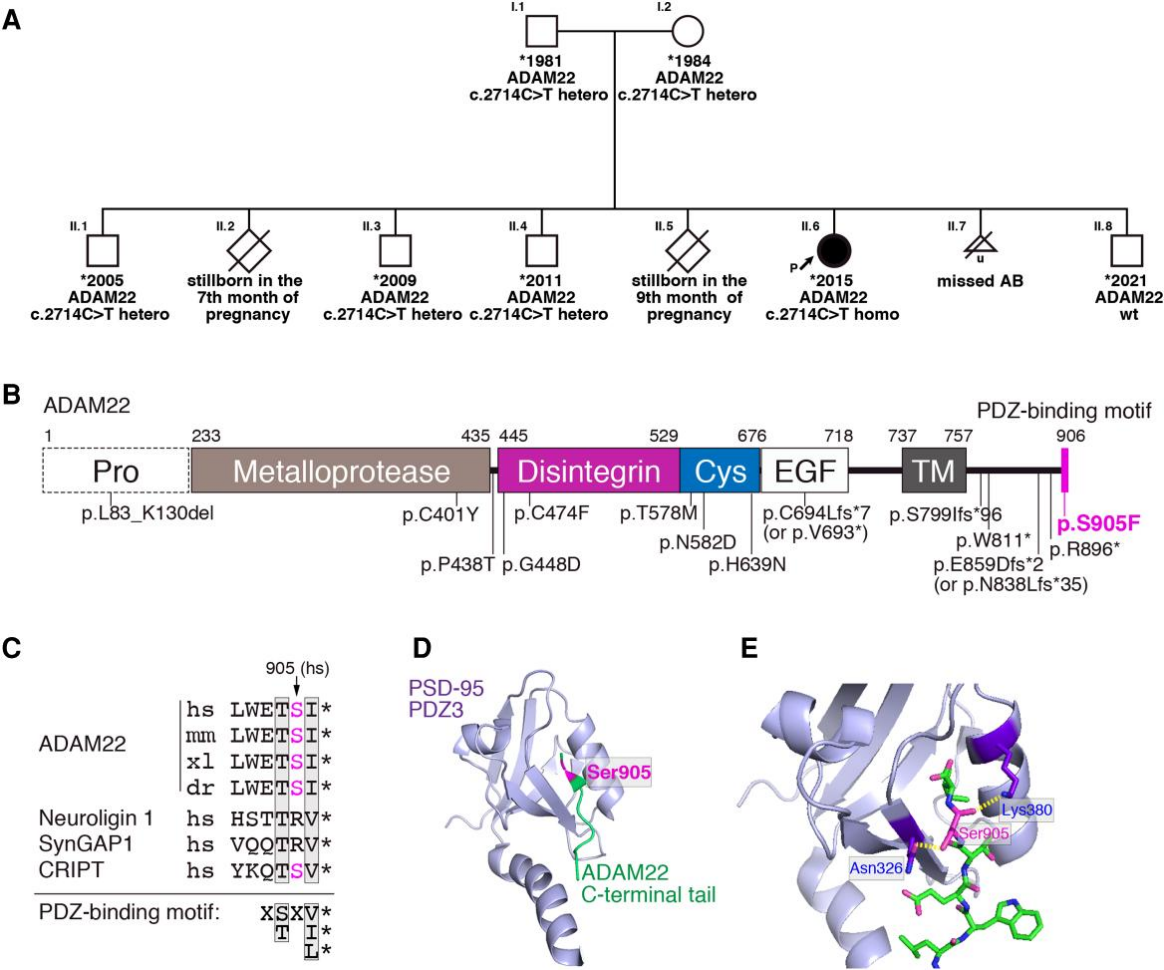
**Genetic study and structural correlates of the *ADAM22* c.2714C > T variant.** (**A)** Pedigree of the family and segregation analysis of the *ADAM22* c.2714C > T variant. (**B)** Domain organization of ADAM22 and location of all reported pathogenic *ADAM22* variants. The p.S905F variant is located in the PDZ-binding motif at the C-terminus (magenta stick). Pro, prodomain; Cys, cysteine-rich domain; EGF, EGF-like domain; TM, transmembrane domain. Note that the ADAM22 has no metalloprotease activity. (**C)** Cross-species sequence alignment of the PDZ-binding motif of ADAM22. hs, *Homo sapiens*; mm, *Mus musculus*; xl, *Xenopus laevis*; dr, *Danio rerio*. The PDZ-biding motifs of neuroligin 1, SynGAP1 and CRIPT that bind to the 3rd PDZ domain of PSD-95 (PDZ3) are aligned. (**D** and **E)** Structure of the complex of the ADAM22 PDZ-binding motif (green) and PSD-95-PDZ3 (purple) (**D)**. Close-up view of the interface between the ADAM22 C-terminal tail and PSD-95-PDZ3 (**E)**. The magenta indicates the position of the Ser905 residue. The yellow dotted lines represent hydrogen bonds between ADAM22 Ser905 residue and PSD-95-PDZ3 domain. PDB accession code, 7CQF.

The mother had recurrent foetal losses (two stillbirths and one spontaneous abortion), other health concerns in parents were not reported. The parents are both of Roma origin. One of the four brothers (II.4, currently 12 years of age) (Fig. 1A) suffers from a behavioural disorder with aggressiveness but no history of seizures. The maternal cousin suffers from epilepsy, but neither medical records nor biological materials are available from this case.

Firstly, the patient presented with the febrile seizures without EEG abnormality at 2 years of age. The seizures reoccurred at the age of 4.5 years and diagnosis of complex focal epilepsy (focal onset impaired awareness seizures) was established. The sleep-deprived EEG showed normal baseline activity and the presence of right frontal irritative focus with signs of secondary generalization. After initiation of the valproic acid treatment, the EEG normalized except for the presence of right-sided sporadic irregular theta waves resulting clinically in seizures ceasing. Brain MRI at the age of 7 years did not reveal any pathological changes. By clinical examination, the hypotonia and hypermobility were noted, no facial dysmorphism or skeletal abnormalities were present, the height and the weight were in normal ranges. No pathological values were observed in biochemical parameters and selective screening for congenital disorders of metabolism.

From the age of 5 years, she was treated for the juvenile rheumatoid arthritis by methotrexate with physical activity limiting arthralgias with good response to antalgics. On last visit at the age of 7 years, the seizures were well controlled by valproate monotherapy with normal EEG record, but the patient continued to have myoclonic jerks on falling asleep and waking up as well as tremor-like episodes with a neuropathic tingling sensation. The intellectual capacity was not evaluated, the patient has expressive speech disorder, hyperkinetic behavioural disorder with features of immaturity and instability. She attends the elementary school from 7 years of age.

### Genetic studies

The patient and her parents underwent WES. A homozygous missense variant in *ADAM22*: NM_021723.5:c.2714C > T (p.Ser905Phe) or NM_001324418.2:c.2888C > T (p.Ser963Phe) was identified in the proband. No other potentially causal variants compatible either with expected autosomal recessive or *de novo* mode of inheritance were identified. Genotyping and segregation analysis identified that only the patient carries the homozygous variant, whereas all other healthy individuals in the family were either heterozygotes or lacked the variant allele (Fig. 1A)

The variant in *ADAM22*: NM_021723.5:c.2714C > T identified in the family is not reported in gnomAD databases. However, in our in-house database of genetic variants of Roma population from the Czech and Slovak Republic, we identified 11 heterozygous carriers of this variant from different families among 213 ethnically related healthy individuals. This is suggestive of a variant allele frequency of ∼5.2% in the Roma population of Czech and Slovak Republic.

### *In silico* analysis

Structural *in silico* characterization revealed that the genetic variant chr7-87825799-C-T (hg19) encodes for a missense variant p.S905F, which localizes in the PDZ-binding domain of ADAM22. Sequence alignment showed that the Ser905 residue of ADAM22 is conserved among representative vertebrates (Fig. 1B and C). Although the S905 does not seem to be a critical residue based on the consensus PDZ-binding motif (−^903^E-T-*S*-I-COOH for X-S/T-*X*-V/I/L-COOH), recent structural analysis (PDB ID, 7CQF)^8^ revealed that the Ser905 of ADAM22 forms hydrogen bonds with Asn326 and Lys380 of the third PDZ domain of PSD-95 (referred to as PDZ3) (Fig. 1D and E). The similar hydrogen bonds observed between ADAM22 and PSD-95 are not apparent in the complex of the CRIPT PDZ ligand and PSD-95-PDZ3 (PDB ID, 1BE9).

### Functional studies

We then examined the effect of the p.S905F variant on binding of ADAM22 to PSD-95 and LGI1. For functional studies and comparison, we also included two previously reported constructs: the ADAM22ΔC5 causing lethal epilepsy of hippocampal origin in mice^8^ and the C401Y described in the patient with severe infantile-onset progressive encephalopathy.^14,16^

**Figure 2:**
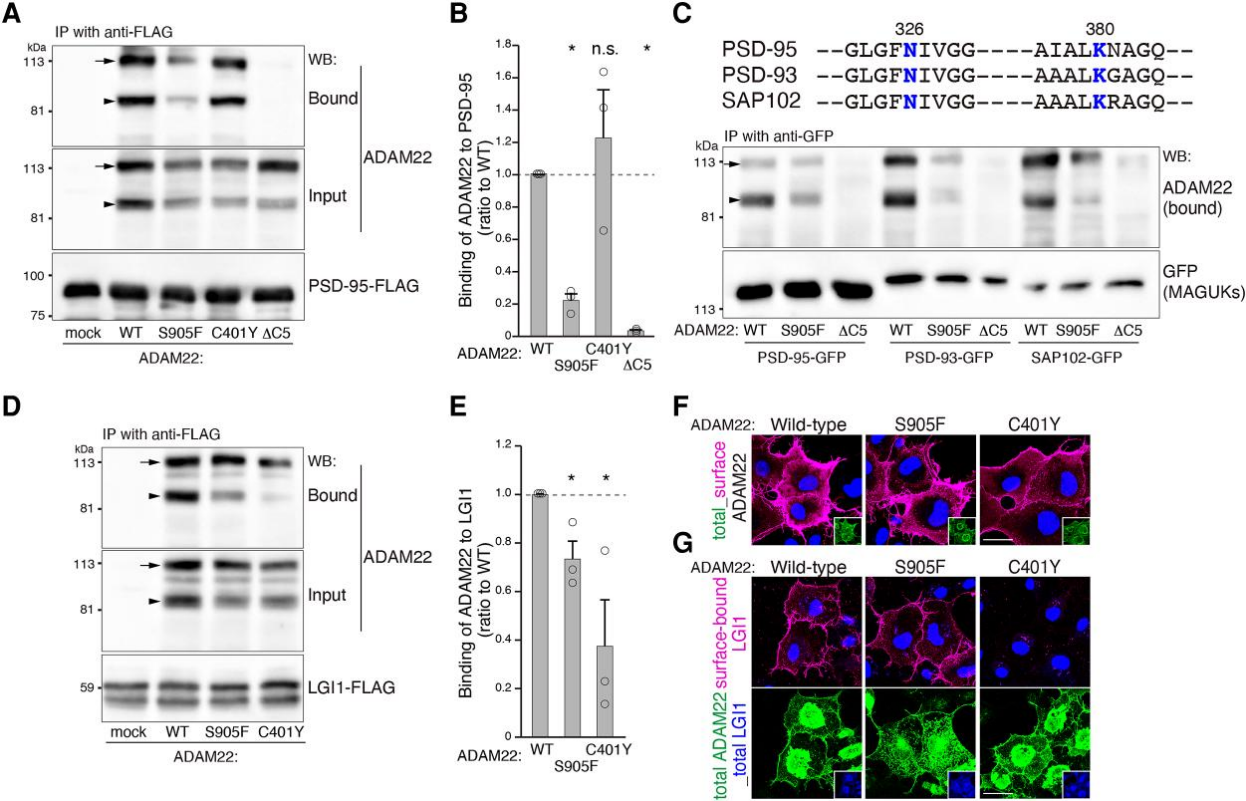
**Functional studies of the ADAM22 protein with S905F variant.** (**A** and **B)** The interaction of ADAM22 S905F with PSD-95 is significantly reduced. Indicated cDNAs for *ADAM22* variants and *PSD-95*–FLAG were co-transfected into HEK293T cells and PSD-95–FLAG was immunoprecipitated (IP). ADAM22 and PSD-95 were detected by western blotting (WB). ADAM22ΔC5, lacking the C-terminal PDZ-binding motif, is used as a control.^8^ Arrows and arrowheads indicate the positions of immature and mature forms of ADAM22, respectively. The immature form of ADAM22 is often observed in overexpressed cells. *P* values were determined by Kruskal–Wallis with *post hoc* Steel test. *P < 0.05; n.s., not significant; *n* = 3 experiments. Results are shown as means ± SE. WT, wild-type. See Supplementary material for uncropped blots for **A**. (**C)** The S905F variant reduces the binding of ADAM22 to MAGUK proteins including PSD-95, PSD-93 and SAP102 in HEK293T cells. Conserved Asn and Lys residues are shown in blue. (**D** and **E)** ADAM22 S905F binds to LGI1. ADAM22 C401Y is used as a control, which shows the reduced binding to LGI1.^7,14^  *P* values are determined by Kruskal–Wallis with *post hoc* Steel test. *P < 0.05; *n* = 3 experiments. Results are shown as means ± SE. Immature ADAM22 (arrows) seems to non-specifically bind to LGI1 under the overexpressed conditions. In the brain, we do not detect such an immature form of ADAM22. See Supplementary material for uncropped blots for **C** and **D**. (**F** and **G)** ADAM22 S905F is expressed at the cell surface and binds to LGI1. Cell-surface-expressed ADAM22 (F, magenta) and cell-surface-bound LGI1 (**G**, magenta) were live-labelled. After fixation and permeabilization of the cells, expressed ADAM22 (total) was stained (green). Nuclear DNA was stained by Hoechst 33342 (**F** and upper panel in **G**, blue). ADAM22 S905F is expressed on the cell surface and binds to LGI1 as the wild-type. When ADAM22 C401Y is expressed, no cell-surface-bound LGI1 is detected. Regions outlined with white squares are total expression of ADAM22 (**F)** and LGI1–FLAG (lower panel in **G**, blue). Bars: 20 μm.

When ADAM22 was co-expressed together with PSD-95 in HEK293T cells, both wild-type ADAM22 and C401Y variant were efficiently co-immunoprecipitated with PSD-95, whereas the binding of ADAM22ΔC5 lacking the PDZ-binding motif to PSD-95 was abolished (Fig. 2A and B). Under these conditions, the interaction of ADAM22 S905F with PSD-95 was reduced to ∼20% compared to wild-type ADAM22, indicating a critical role of ADAM22 Ser905 residue in PSD-95 binding. The Asn326 and Lys380 residues of the PSD-95-PDZ3 are common to other PSD-95 family membrane-associated guanylate kinases (MAGUKs) including PSD-93 and SAP102 (Fig. 2C, upper). Consistently, the binding of ADAM22 S905F with either PSD-93 or SAP102 was reduced to ∼20% compared to the wild-type protein, indicating that the S905F variant affects the binding of ADAM22 to MAGUK family proteins.

Next, we investigated the effect of ADAM22 S905F variant on LGI1-binding. An immunoprecipitation assay from co-transfected HEK293T cells showed that co-immunoprecipitation of ADAM22 S905F with LGI1 was reduced to ∼75% compared to the wild-type protein (Fig. 2D and E). In contrast, the variant of the Cys401 residue that plays a supportive role in the LGI1-binding interface formation reduced the ADAM22 binding to LGI1 to ∼40% as previously reported.^7,14^ Consistently, when cell-surface-expressed ADAM22 was ‘live-stained’ with an antibody to the extracellular epitope of ADAM22, both ADAM22 S905F and wild-type ADAM22 were efficiently detected on the cell-surface (Fig. 2F) bound to the LGI1 ligand (Fig. 2G). These results indicate that ADAM22 S905F protein is normally expressed and binds to the LGI1 ligand on the cell surface, but shows reduced binding to MAGUKs.

## Discussion

We report the case of a girl with early-onset focal epilepsy, delayed psychomotor development and behavioural disorder carrying a novel homozygous pathogenic variant in *ADAM22*. The variant is of particular interest for clinical geneticists, as we identified it in heterozygous state in 11/213 (5.2%) individuals of Roma origin from the Czech and Slovak Republic. This carrier frequency in genetically isolated and homogenous population indicates a possible founder effect and implicates that high proportion of epilepsy in Roma patients may result from homozygosity for this particular variant. Another variant with possible founder effect disrupting interaction with MAGUKs was reported in three unrelated Middle-Eastern families.^15,16^

The patient presented with milder phenotype, with later age at seizures onset (2 years), good response to anti-epileptics (controlled by valproate monotherapy) and normal MRI, as compared to earlier age of onset (0–18 months), persistent pharmacoresistant seizures and MRI abnormalities observed in the patients with previously reported pathogenic variants of *ADAM22*.^14,16^

The milder clinical presentation probably reflects particularly different pathogenetic mechanism of the p.S905F variant. Most of the pathogenic missense variants of ADAM22 that were identified in previous studies are located in the extracellular domain (Fig. 1B), and lead to reduced/diminished biosynthesis and maturation of the ADAM22 protein and/or reduced/completely abrogated binding of ADAM22 to LGI1. The establishment of ADAM22–LGI1 complex is indispensable for normal ADAM22 function and its significant reduction in LGI1-binding capacity leads to epileptic encephalopathy.^16^ In contrast, only three pathogenic variants located in the intracellular domain were reported (Q859DfsTer2, pW811* and R896*), and lead to complete abolition of the interaction with PSD-95, causing complicated neurological symptoms: including epilepsy, intellectual disability, sleep disturbance, hyperphagia, attention deficit hyperactivity disorder (ADHD) and some autistic-like features.^15,16^ Consistently, loss of the ADAM22 C-terminal PDZ-binding motif (−W*ETSI*, ΔC5) in mice causes lethal epilepsy of hippocampal origin.^8^ The ADAM22 p.S905F variant we identified here is novel in that it is only ‘partially’ affecting conserved PDZ-binding motif of ADAM22. Our functional studies indicate that ADAM22 S905F maturates normally and its binding to LGI1 is not as much affected. Instead, ADAM22 S905F shows ‘reduced’ binding capacity (∼20% of wild-type protein) to intracellular MAGUKs (PSD-95, PSD-93 and SAP102) (Fig. 2A–C). The milder phenotype of patient with homozygous p.S905F variant may represent the mildest type of disease spectrum caused by ADAM22 pathogenic variants ranging from fully inactivating variants to variants retaining residual LGI1- and/or MAGUK-binding capacity.

The ADAM22 p.S905F variant is located just inside the PDZ-binding domain (–W*E*T***S***I–COOH), but the serine residue does not seem to be a critical residue judging from the consensus sequence for the PDZ-binding motif (X-S/T-X-V/I/L-COOH). However, the significant reduction of binding capacity to MAGUKs suggests that the p.S905F variant in ADAM22 represents a partial loss-of-function variant and the corresponding bi-allelic (homozygous) variant is causative of brain disorders in the patient, akin to cases of bi-allelic variants due to the compound heterozygosity or homozygosity of *ADAM22* variants in progressive encephalopathy.^14-16^ The residual level of interaction capacity may thus explain milder symptoms of the patient presented in this study. The phenotypic features in our patient also does not fully corresponds to epileptic encephalopathy as reported in previous studies with other bi-allelic *ADAM22* variants,^14-16^ rather represent epilepsy with neurodevelopmental disorder and extend the phenotype towards milder spectrum.

The patient with a homozygous ADAM22 S905F variant also suffers from polyarticular form of juvenile idiopathic arthritis. It was reported that bi-allelic variants in *LGI4*, a member of the LGI family proteins, cause arthrogryposis multiplex congenita (AMC) with peripheral nerve hypomyelination,^20,21^ and *Lgi4* knockout mice exhibit an AMC-like forelimb defect.^22-24^ Given that ADAM22 serves also as a receptor for LGI4 in the peripheral nerve system, the symptom of ‘idiopathic arthritis’ observed in the patient may be related to loss of function of ADAM22 in the peripheral nerve system.

It was shown that ∼10% of ADAM22 and ∼50% of LGI1 protein amounts could suppress lethal spontaneous seizures in mice.^25^ We show that the amount of the p.S905F ADAM22–LGI1 complex on the cell surface is comparable to wild-type cells, indicating that LGI1–ADAM22 interaction and complex stability is not disturbed. It can be speculated that stabilization of ADAM22–LGI1 complex to cell surface via 14-3-3 proteins should not be affected as PSD-95 and 14-3-3 bind to ADAM22 without competition.^25^ The main pathogenic mechanism in a patient harbouring ADAM22 S905F variant thus seem to result from insufficient interaction of ADAM22–MAGUKs leading to reduced synaptic AMPA receptor functions^9,12^ and reduced axonal Kv_1_ channel function.^3^

The results of functional studies of ADAM22 S905F variant reconfirm the essential role of ADAM22–MAGUK interaction^8^ and reveal that ∼20% of the residual ADAM22–MAGUK interaction is not sufficient to suppress the clinical symptoms. Given that the MAGUK–ADAM22 linkage plays an essential role in hippocampal long-term potentiation,^10^ a widely accepted cellular model for learning and memory, the ADAM22–MAGUK interaction could become a therapeutic target for synaptic disorders, such as intellectual disability and epilepsy.

## Supplementary Material

fcad295_Supplementary_Data

## Data Availability

Because of the rarity of the disorder, individual participant data beyond that reported in this article will not be shared, to safeguard patient privacy. Other experimental data are available from the corresponding authors upon reasonable request.
