# Supplementary figures and images for: ADAM22 ethnic-specific variant reducing binding of membrane-associated guanylate kinases causes focal epilepsy and behavioural disorder
Source: Brain Commun. 2023 Oct 27;5(6):fcad295. doi: 10.1093/braincomms/fcad295 (PMC10636567; doi:10.1093/braincomms/fcad295)

### Related to Fig. 2A

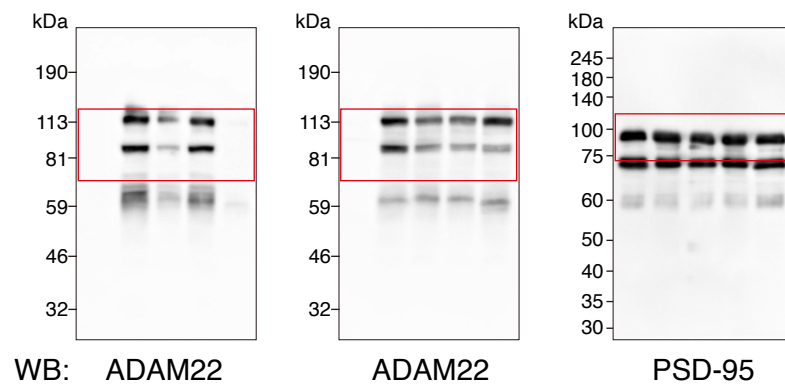

### Related to Fig. 2C

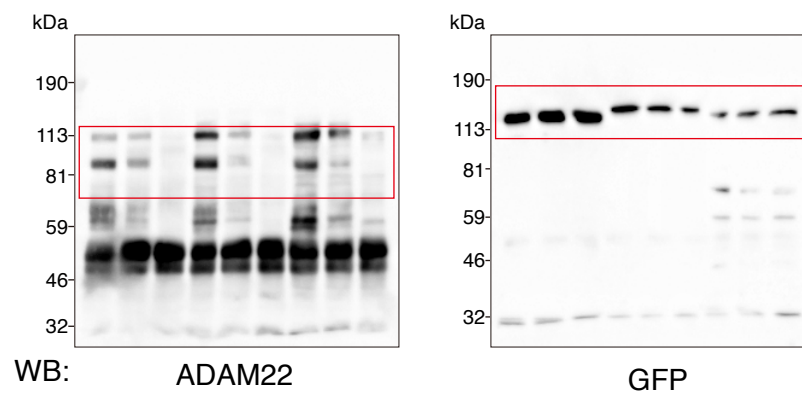

### Related to Fig. 2D

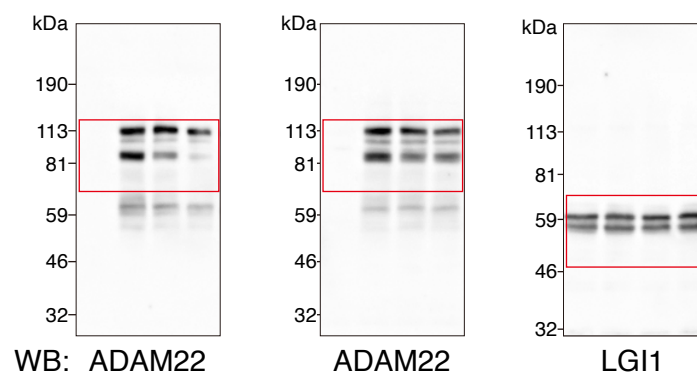

Supplement: fcad295_Supplementary_Data [file fcad295_supplementary_data.pdf]
